# Supplementary material for: Cell-specific responses of Anopheles gambiae fat body to blood feeding and infection at single-nuclei resolution
Source: Nat Commun. 2026 Feb 24;17:3119. doi: 10.1038/s41467-026-69806-1 (PMC13043729; doi:10.1038/s41467-026-69806-1)
Supplement: Supplementary file 1 — Supplementary Information [file 41467_2026_69806_MOESM1_ESM.pdf]

Cell-specific responses of *Anopheles gambiae* fat body to blood feeding and infection at Single-nuclei resolution

Authors: Stephanie Serafim de Carvalho[1], Colton McNinch[2], Ana-Beatriz F. Barletta[1], Carolina Barillas-Mury[1*]

Affiliations

[1] Laboratory of Malaria and Vector Research, National Institutes of Allergy and Infectious Diseases, National Institutes of Health; Rockville, Maryland, 20852, USA.

[2] Bioinformatics and Computational Biosciences Branch, Office of Cyber Infrastructure and Computational Biology, National Institute of Allergy and Infectious Diseases, National Institutes of Health; Bethesda, Maryland, 20892, USA.

*Corresponding authors. Email: cbarillas@niaid.nih.gov

The PDF file includes:

Supplementary Figures 1 to 9

Other supporting materials for this manuscript:

Supplementary Data 1 to 8
Source Data

Supplementary Figures

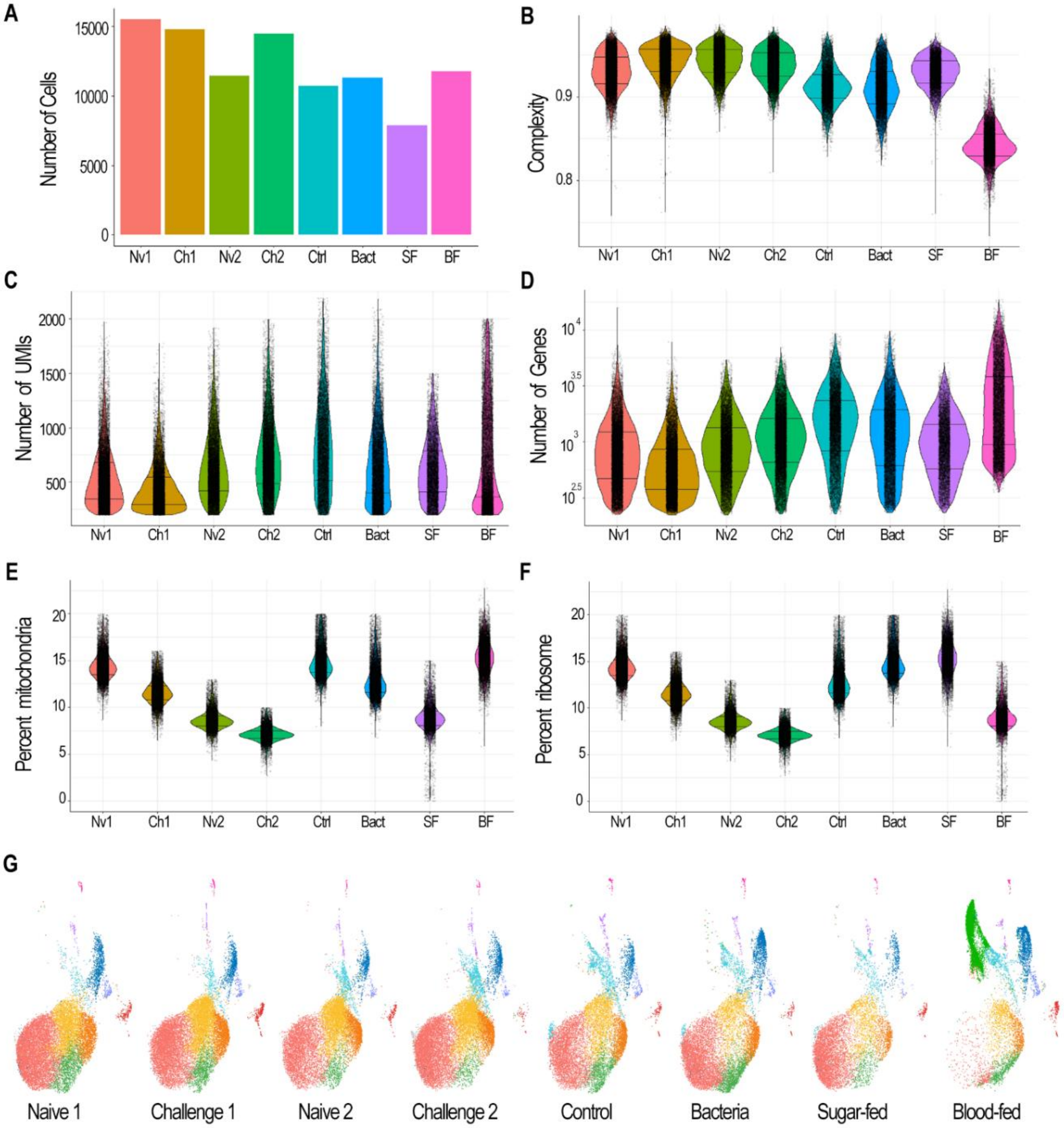

Supplementary Figure 1. Quality control metrics and clustering analysis of *Anopheles gambiae* abdominal body wall single-nucleus RNA-seq data. (A) Total number of cells captured per sample. (B) Distribution of complexity per sample, represented as log10GenesPerUMI. (C) Total number of UMIs per sample. (D) Number of detected genes (features) per sample. (E) Percentage of mitochondrial gene expression per sample. (F) Percentage of ribosomal gene expression per sample. (G) UMAP visualization of integrated abdominal body wall nuclei, shown separately for each sample, at clustering resolution 0.3. Nv: Naïve, Ch: *P. berghei*-challenged, Ctrl: control-PBS-injected, Bact: Bacteria-injected, SF: Sugar-fed, BF: Blood-fed.

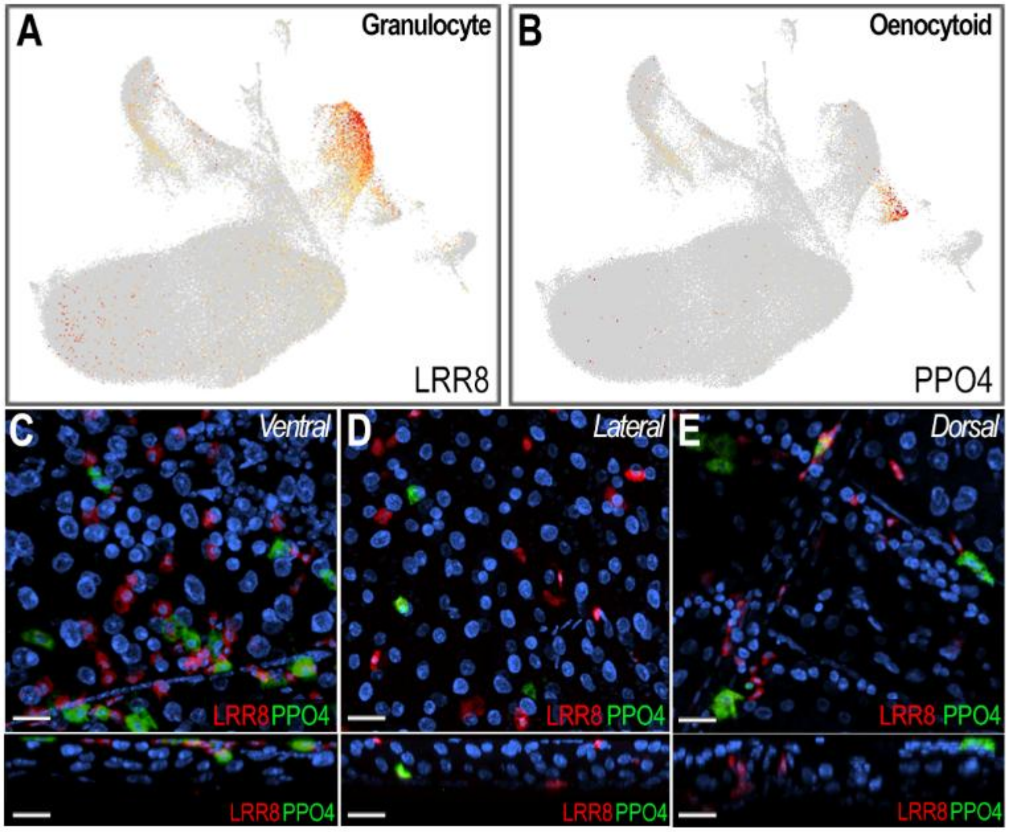

Supplementary Figure 2. Localization and characterization of fat body-associated hemocytes. (A) Feature plot showing expression of Leucine-rich repeat 8 (*LRR8*; *AGAP004017*), a marker of the granulocytes (cluster Hm1). (B) Feature plot showing expression of prophenoloxidase 4 (*PPO4*; *AGAP004981*), a marker of the oenocytoids (cluster Hm2). (C-E) RNA *in situ* hybridization of *LRR8* and *PPO4* on ventral (C), lateral (D), and dorsal (E) regions of abdominal fat body tissue. (C-E, bottom) Corresponding side views of each region (N=2, 10-15 tissues). *LRR8* is shown in red, *PPO4* in green, and nuclei in blue. Scale bar: 20µm.

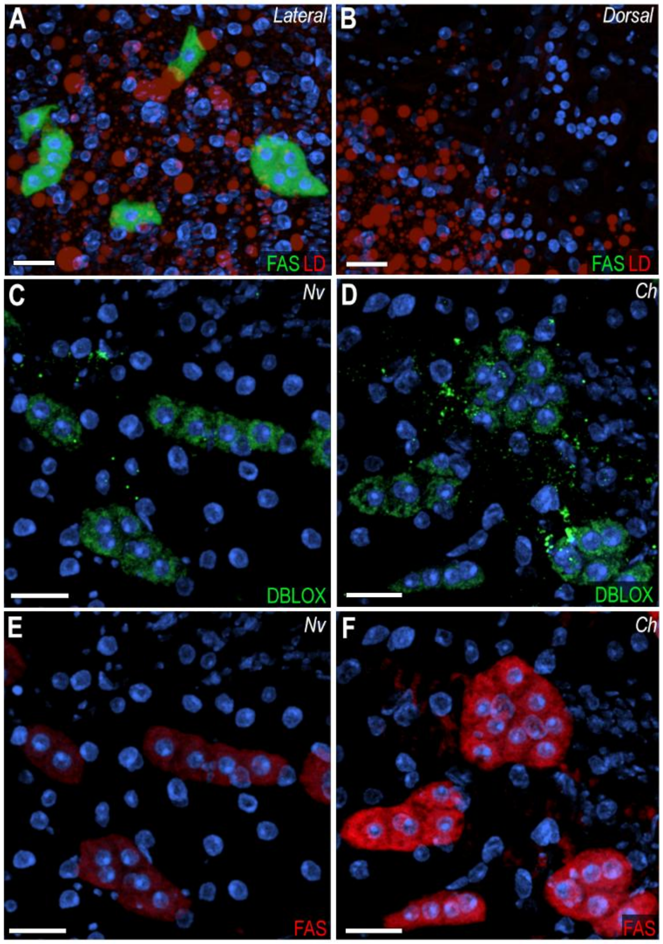

Supplementary Figure 3. Characterization of oenocytes and their response to immune priming. (A-B) RNA *in situ* hybridization of Fatty acid synthase (*FAS*; *AGAP001899*), a marker of oenocytes, on the lateral (A) and dorsal (B) sides of the abdominal body wall (N=2, 10-15 tissues). FAS is shown in green, lipid droplet (LD) in red, and nuclei in blue. (C-H) RNA *in situ* hybridization of *FAS* and double peroxidase (*DBLOX*) in ventral abdominal fat body tissue from Naïve (Nv) (C, E, G) and *Plasmodium berghei*-challenge (Ch) (D, F, H) conditions (N=2, 10-15 tissues). *FAS* is stained in red, DBLOX in green, and nuclei in blue. Scale bar: 20µm.

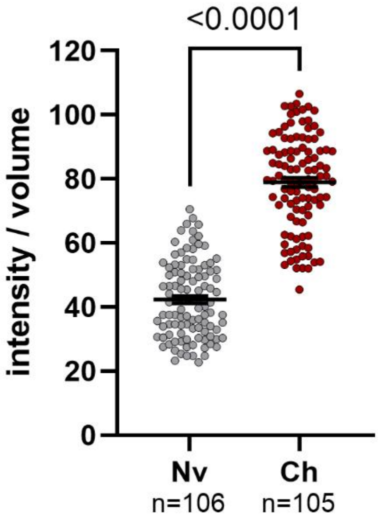

Supplementary Figure 4. Quantification of *FAS* RNA FISH fluorescence intensity in oenocytes from naïve and *Plasmodium berghei* challenged mosquitoes. Mean fluorescence intensity per oenocyte voxel was quantified from RNA FISH images obtained from naïve and primed females (6–7 fields per condition). Each point represents the average voxel-level intensity for each oenocyte cluster. Nv: Naïve, Ch: *P. berghei*-challenged. Bars indicate mean ± SEM. Two-tailed Mann-Whiteney test, p<0.001. Source data are provided as a Source Data file.

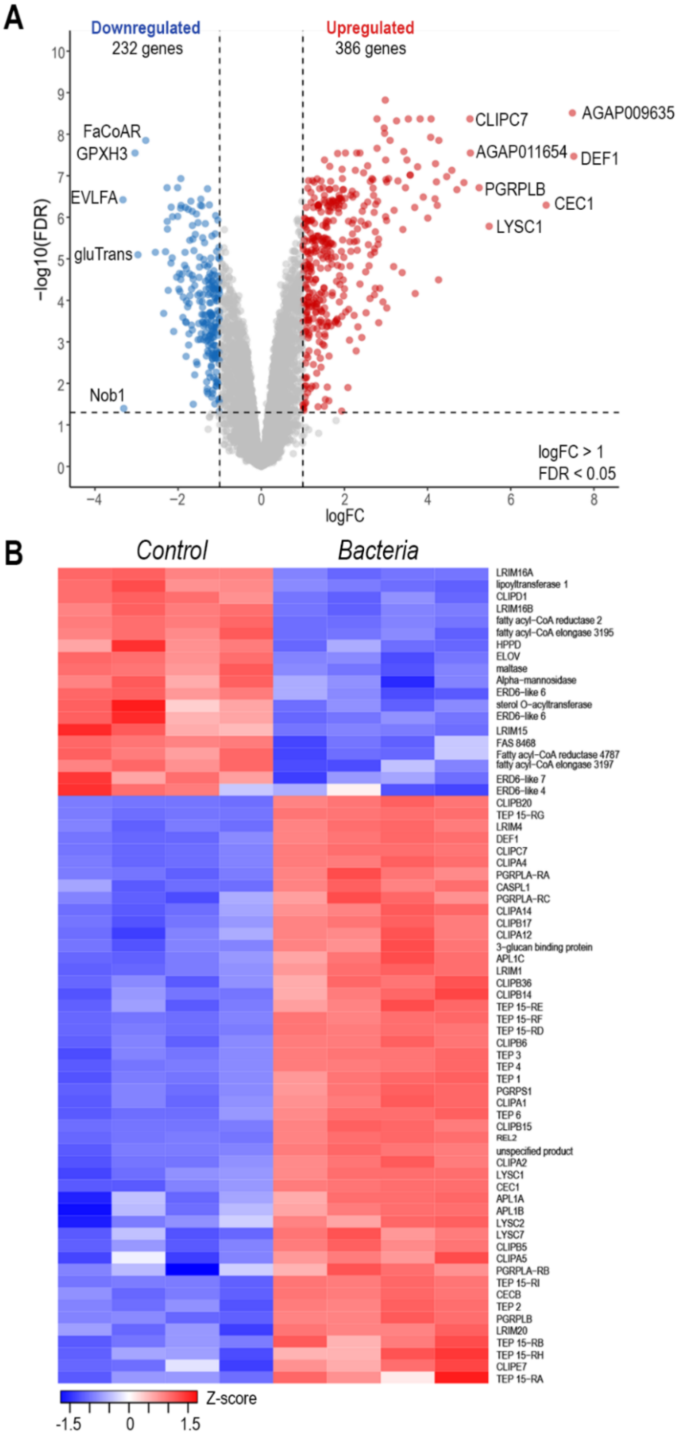

Supplementary Figure 5. Effect of bacterial infection on mRNA expression. (A) Volcano plot and (B) heatmap showing differentially expressed genes in bacteria-infected mosquito fat body tissue compared with PBS-injected control, filtered for $\log_2$ Fold Change > 1 and FDR < 0.05 (N=4, 10-15 tissues). Blue dots represent downregulated genes, and red dots represent upregulated genes. The complete list of differentially expressed genes is listed in Supplementary Table 5.

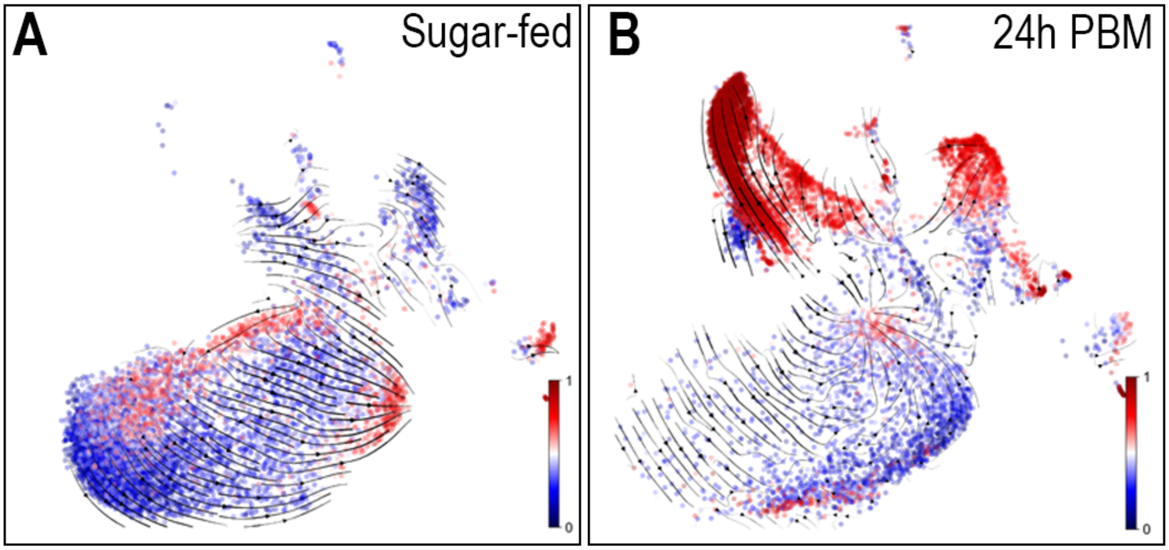

Supplementary Figure 6. Transcriptional shifts between sugar-fed and blood-fed samples. RNA-velocity analysis of nuclei collected from (A) sugar-fed and (B) blood-fed mosquitoes 24h post-blood meal (PBM). Arrows indicate the predicted transcriptional trajectories of the cells. The red-to-blue color scale indicates the relative abundance of unspliced transcripts, with warmer colors reflecting higher enrichment.

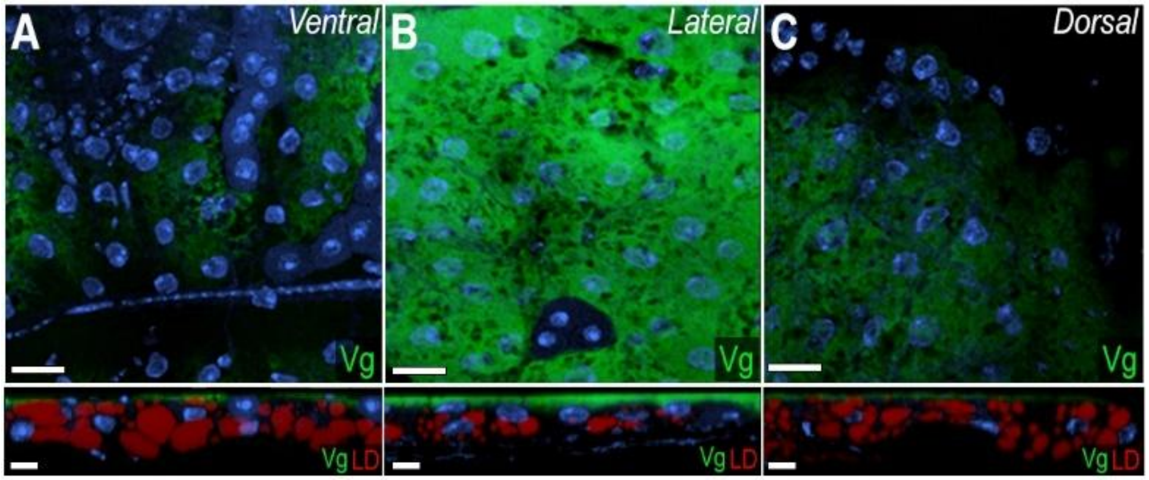

Supplementary Figure 7. Vitellogenin mRNA expression pattern on abdominal fat body tissue. (A-C) RNA *in situ* hybridization of Vitellogenin (*Vg*; *AGAP004203*) in the abdominal fat body of blood-fed females, shown on the ventral (A), lateral (B), and dorsal (C) sides. (A-C, bottom) Corresponding side views of each region (N=3, 10-15 tissues). *Vg* is shown in green, lipid droplets (LD) in red, and nuclei in blue. Scale bar: 20µm.

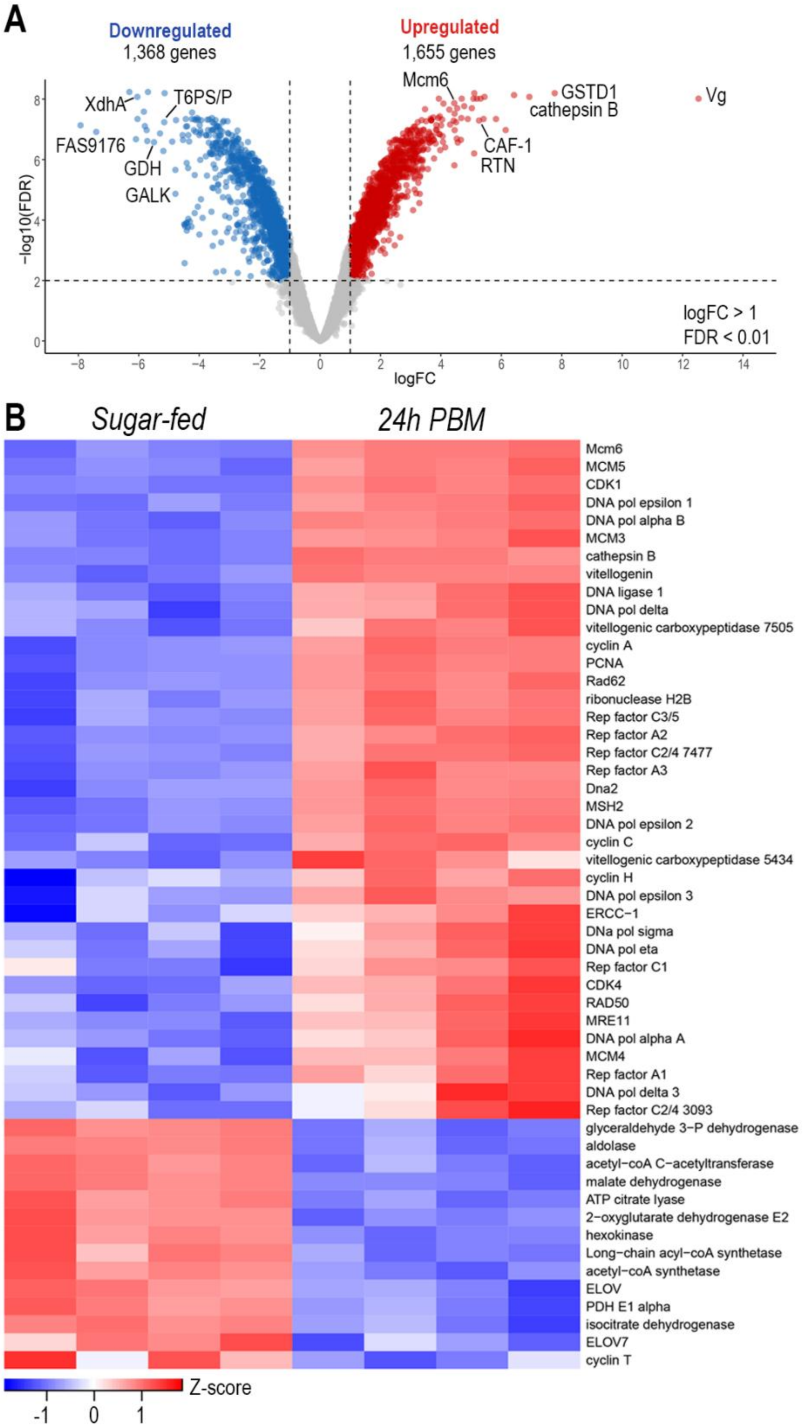

Supplementary Figure 8. Effect of blood feeding on mRNA expression. (A) Volcano plot and (B) Heatmap showing differentially expressed genes in blood-fed (24h) mosquito fat body tissue compared with sugar-fed, filtered for log_2 Fold Change > 1 and FDR < 0.01 (N=4, 10-15 tissues). Blue dots represent downregulated genes, and red dots represent upregulated genes. The complete list of differentially expressed genes is listed in Supplementary Table 8.

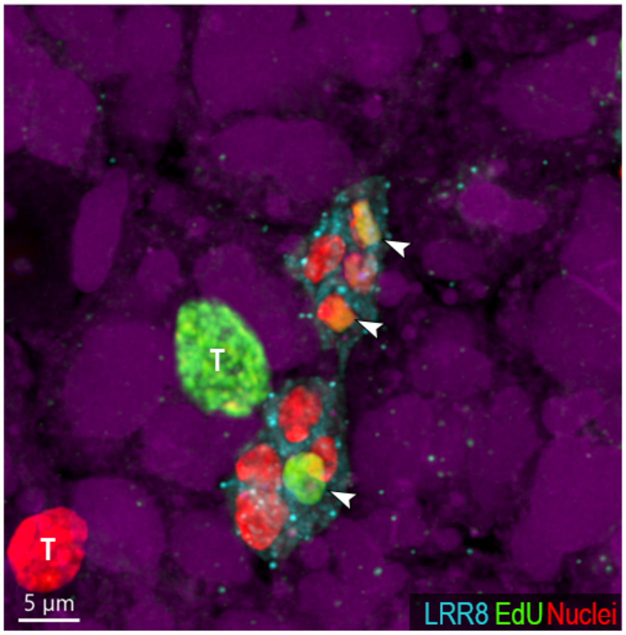

Supplementary Figure 9. LRR8 expression pattern and EdU staining on hemocytes of abdominal fat body tissue. Immunofluorescence assay (IFA) of Leucin-rich repeat protein 8 (*LRR8*; *AGAP004017*) in the abdominal fat body of blood-fed females (N=1, 10-15 tissues). LRR8 is shown in cyan, nuclei in red, 5-ethynyl-2'-deoxyuridine (EdU) in green and tissue structure in purple. T indicates trophocyte nuclei and arrowheads indicate *LRR8* and EdU positive cells. Scale bar: 5µm.
