# Supplementary material for: Cell-specific responses of Anopheles gambiae fat body to blood feeding and infection at single-nuclei resolution
Source: Nat Commun. 2026 Feb 24;17:3119. doi: 10.1038/s41467-026-69806-1 (PMC13043729; doi:10.1038/s41467-026-69806-1)
Supplement: Supplementary file 2 — Description of Additional Supplementary Files [file 41467_2026_69806_MOESM2_ESM.pdf]

Description of Additional Supplementary Files

Supplementary Data 1. Marker genes for each *Anopheles gambiae* fat body cluster. Marker genes were identified using a two-sided Wilcoxon rank-sum test comparing each cluster with all other cells. P values were adjusted for multiple comparisons using the Benjamini–Hochberg false discovery rate (FDR) correction. For each marker gene, the table reports the corresponding cluster identifier, average $\log_2$ fold change (Avg $\log_2FC > 0.5$), adjusted P value (P Val adj < 0.05), percentage of cells expressing the gene within the cluster, and VectorBase AGAP accession number.

Supplementary Data 2. Distribution of captured single cells across samples and clusters. Proportion of cells assigned to each cluster per sample.

Supplementary Data 3. Differentially expressed transcripts in naïve and *Plasmodium berghei*-challenged *Anopheles gambiae* fat body. Differential expression analysis was performed on bulk RNA-seq data using edgeR, applying a two-sided quasi-likelihood F-test to compare naïve and *P. berghei*–challenged samples. P values were adjusted for multiple comparisons using the Benjamini–Hochberg false discovery rate (FDR) correction. Transcripts with an absolute $\log_2$ fold change > 0.5 and FDR < 0.05 are reported.

Supplementary Data 4. Differentially expressed genes in granulocytes (Hm1) from naïve and *Plasmodium berghei*-challenged *Anopheles gambiae*. Differential expression analysis was performed on single-nucleus RNA-seq data from granulocytes (Hm1) and oenocytes (Oe) isolated from fat body tissue of naïve and *P. berghei*–challenged *An. gambiae*. Differentially expressed genes were identified using a two-sided Wilcoxon rank-sum test. P values were adjusted for multiple comparisons using the Benjamini–Hochberg false discovery rate (FDR) correction. Genes with an average $\log_2$ fold change > 0.5 or < -0.5 and adjusted P value (P Val adj < 0.05) are reported.

Supplementary Data 5. Differentially expressed transcripts in control and bacteria-injected *Anopheles gambiae* fat body. Differential expression analysis was performed on bulk RNA-seq data using edgeR, applying a two-sided quasi-likelihood F-test to compare control and bacteria-injected samples. P values were adjusted for multiple comparisons using the Benjamini–Hochberg false discovery rate (FDR) correction. Transcripts with an absolute $\log_2$ fold change > 1 and FDR < 0.05 are reported.

Supplementary Data 6. Differentially expressed genes in trophocytes (T1-T4), hemocytes (Hm1 and Hm2), epidermal cells (EC), and pericardial cells (PC) from control and bacteria-injected *Anopheles gambiae*. Differential expression analysis was performed on single-nucleus RNA-seq data from trophocytes (T1–T4), hemocytes (Hm1 and Hm2), epidermal cells (EC), and pericardial cells (PC) isolated from fat body tissue of control and bacteria-injected *An. gambiae*. Differentially expressed genes were identified using a two-sided Wilcoxon rank-sum test. P values were adjusted for multiple comparisons using the Benjamini–Hochberg false discovery rate (FDR) correction. Genes with an average $\log_2$ fold change > 1 or < −1 and adjusted P value (P Val adj < 0.05) are reported.

Supplementary Data 7. Differentially expressed genes in T1 and T5 trophocytes from sugar-fed and 24-hour post-blood meal *Anopheles gambiae*. Differential expression analysis was performed on single-nucleus RNA-seq data from T1 and T5 trophocytes isolated from fat body tissue of sugar-fed and 24-h post-blood-meal *An. gambiae*. Differentially expressed genes were identified using a two-sided Wilcoxon rank-sum test. P values were adjusted for multiple comparisons using the Benjamini–Hochberg false discovery rate (FDR) correction. Genes with an average $\log_2$ fold change > 1 or < −1 and adjusted P value (P Val adj < 0.05) are reported.

Supplementary Data 8. Differentially expressed transcripts in sugar-fed and 24-

hour post-blood meal *Anopheles gambiae* fat body. Differential expression analysis was performed on bulk RNA-seq data using edgeR, applying a two-sided quasi-likelihood F-test to compare sugar-fed and 24-h post-blood-meal samples. P values were adjusted for multiple comparisons using the Benjamini–Hochberg false discovery rate (FDR) correction. Transcripts with an absolute $\log_2$ fold change > 1 and FDR < 0.01 are reported.
