# Supplementary material for: Cell-specific responses of Anopheles gambiae fat body to blood feeding and infection at single-nuclei resolution
Source: Nat Commun. 2026 Feb 24;17:3119. doi: 10.1038/s41467-026-69806-1 (PMC13043729; doi:10.1038/s41467-026-69806-1)
Supplement: Supplementary file 11 — Reporting Summary [file 41467_2026_69806_MOESM11_ESM.pdf]

nature portfolio

Corresponding author(s): Carolina Barillas-Mury

Last updated by author(s): Dec 11, 2025

Reporting Summary

Nature Portfolio wishes to improve the reproducibility of the work that we publish. This form provides structure for consistency and transparency in reporting. For further information on Nature Portfolio policies, see our Editorial Policies and the Editorial Policy Checklist.

Statistics

For all statistical analyses, confirm that the following items are present in the figure legend, table legend, main text, or Methods section.

n/a	Confirmed	
☐	☒	The exact sample size (*n*) for each experimental group/condition, given as a discrete number and unit of measurement
☐	☒	A statement on whether measurements were taken from distinct samples or whether the same sample was measured repeatedly
☐	☒	The statistical test(s) used AND whether they are one- or two-sided<br>*Only common tests should be described solely by name; describe more complex techniques in the Methods section.*
☐	☒	A description of all covariates tested
☐	☒	A description of any assumptions or corrections, such as tests of normality and adjustment for multiple comparisons
☐	☒	A full description of the statistical parameters including central tendency (e.g. means) or other basic estimates (e.g. regression coefficient) AND variation (e.g. standard deviation) or associated estimates of uncertainty (e.g. confidence intervals)
☐	☒	For null hypothesis testing, the test statistic (e.g. F, t, r) with confidence intervals, effect sizes, degrees of freedom and P value noted<br>*Give P values as exact values whenever suitable.*
☒	☐	For Bayesian analysis, information on the choice of priors and Markov chain Monte Carlo settings
☒	☐	For hierarchical and complex designs, identification of the appropriate level for tests and full reporting of outcomes
☒	☐	Estimates of effect sizes (e.g. Cohen's *d*, Pearson's *r*), indicating how they were calculated

Our web collection on statistics for biologists contains articles on many of the points above.

Software and code

Policy information about availability of computer code

Data collection	Leica Application Suite X (LAS X) software was used to capture microscopy images.
Data analysis	For single-nucleus RNA-seq analysis: Cell ranger software (v. 7.0.0) was used to build a custom reference and perform alignment and quantification to generate filtered gene-barcode matrices. DecontX algorithm from celda R package (version 1.20.0) was used to remove contamination from ambient RNA. Seurat R package (v.4.2.1) was used for quality control and cell filtering, normalization, integration, clustering analysis, and gene marker discovery. RNA velocity analysis was performed with velocyto (v.0.17.15) R package and projected into UMAP embeddings using scVelo (v.0.2.5) R package. For bulk RNA-seq analysis: raw read quality was checked using FastQC (v.0.12.0). RSEM (v1.3.3) was used for read alignment and transcript quantification. edgeR R package (v. 3.19) was used to perform pairwise differential expression analysis. For image processing and quantification: Imaris (v.10.0.0) software was used for image processing and quantification of in situ hybridization images. Statistical analysis: GraphPad prism (v. 10) was used to perform statistical analysis of in situ hybridization pixel quantification.

For manuscripts utilizing custom algorithms or software that are central to the research but not yet described in published literature, software must be made available to editors and reviewers. We strongly encourage code deposition in a community repository (e.g. GitHub). See the Nature Portfolio guidelines for submitting code & software for further information.

Data

Policy information about availability of data

All manuscripts must include a data availability statement. This statement should provide the following information, where applicable:

- Accession codes, unique identifiers, or web links for publicly available datasets
- A description of any restrictions on data availability
- For clinical datasets or third party data, please ensure that the statement adheres to our policy

Data Availability
Raw fastq files from single nucleus and bulk RNA sequencing are available the SRA database under the Bioproject number PRJNA1288431 (https://dataview.ncbi.nlm.nih.gov/object/PRJNA1288431?reviewer=he84ttfsjqcmttfp3ccg035vi8). All other relevant data are provided within the paper and its Supplementary files. Source data are provided with this paper.

Code Availability
The code supporting the findings of this study has been deposited in Zenodo and is accessible via the DOI: 10.5281/zenodo.17886096. The repository contains all analysis scripts and documentation required to reproduce the results.

Research involving human participants, their data, or biological material

Policy information about studies with human participants or human data. See also policy information about sex, gender (identity/presentation), and sexual orientation and race, ethnicity and racism.

Reporting on sex and gender	N/A
Reporting on race, ethnicity, or other socially relevant groupings	N/A
Population characteristics	N/A
Recruitment	N/A
Ethics oversight	N/A

Note that full information on the approval of the study protocol must also be provided in the manuscript.

Field-specific reporting

Please select the one below that is the best fit for your research. If you are not sure, read the appropriate sections before making your selection.

☒ Life sciences ☐ Behavioural & social sciences ☐ Ecological, evolutionary & environmental sciences

For a reference copy of the document with all sections, see nature.com/documents/nr-reporting-summary-flat.pdf

Life sciences study design

All studies must disclose on these points even when the disclosure is negative.

Sample size	No sample sizes calculations were performed. Single nuclei sequencing captures thousands of cells per sample, we prioritize biological conditions (N = 8) over technical replicates. For bulk RNA sequencing we collected 3 or 4 independent replicates with pools of 10-15 mosquitoes per sample. This is a commonly used number of samples for this kind of data. For in situ hybridizations and immunofluorescence, the experiments were performed independently twice, using 8-10 individual mosquitoes per experiment.
Data exclusions	No data was excluded
Replication	Single-nucleus RNA sequencing was performed twice for naïve and P. berghei–challenged mosquitoes, and once for the other biological samples. The number of samples was chosen based on the literature, as thousands of nuclei were captured from each sample and the single-cell library kits are costly. Each sample was collected from a pool of 25 mosquitoes.<br>Bulk RNA sequencing experiments were performed with 3 or 4 biological replicates, each consisting of a pool of 10–15 mosquitoes per sample.<br>For microscopy experiments (in situ hybridization and immunofluorescence), we performed two independent experiments using 8–10 individual mosquitoes.
Randomization	We performed pairwise comparisons, therefore randomization was not required.
Blinding	Blinding is not applicable, as single-nucleus and bulk-RNA seq workflow rely on computational analysis that minimize the researcher bias.

Reporting for specific materials, systems and methods

We require information from authors about some types of materials, experimental systems and methods used in many studies. Here, indicate whether each material, system or method listed is relevant to your study. If you are not sure if a list item applies to your research, read the appropriate section before selecting a response.

Materials & experimental systems

n/a	Involved in the study	
☐	☒	Antibodies
☒	☐	Eukaryotic cell lines
☒	☐	Palaeontology and archaeology
☐	☒	Animals and other organisms
☒	☐	Clinical data
☒	☐	Dual use research of concern
☒	☐	Plants

Methods

n/a	Involved in the study	
☒	☐	ChIP-seq
☒	☐	Flow cytometry
☒	☐	MRI-based neuroimaging

Antibodies

Antibodies used	anti-phospho-histone H3 (Ser10), clone 3H10 (Millipore Sigma, MA, USA) 1:1,000 – catalog number: 06-570, lot number: 3113883<br>custom made anti-Leucin rich protein 8 (LRR8) (Pacific Immunology, CA, USA) 1:100 – lot number: PAC-14245, PAC-14246<br>goat anti-rabbit immunoglobulin G (IgG)–Alexa Fluor 555nm (Invitrogen, CA, USA) 1:1,000 – catalog number: A21429, lot number: 2015563
Validation	Antibodies were used for immunofluorescence assays (IFA) at 1:1000 dilution for anti-phospho-histone H3 (https://www.sigmaaldrich.com/US/en/product/mm/06570) and 1:100 dilution for anti-LRR8 antibody. Both antibodies showed a specific staining with minimal to no background. Methodology is described under “5-ethynyl-2'-deoxyuridine (EdU) assay and Immunofluorescence” section.

Animals and other research organisms

Policy information about studies involving animals; ARRIVE guidelines recommended for reporting animal research, and Sex and Gender in Research

Laboratory animals	Female BALB/c mice (Mus musculus) - 3-4 weeks old; Adult female Anopheles gambiae mosquitoes (G3 strain - CDC).
Wild animals	No wild animals were used in this study.
Reporting on sex	All mosquitoes used in this study were females.
Field-collected samples	No field-collected samples were used in this study.
Ethics oversight	Public Health Service Animal Welfare Assurance #A4149-01 guidelines were followed according to the National Institutes of Health Animal (NIH) Office of Animal Care and Use (OACU). These studies were done according to the NIH animal study protocol (ASP) approved by the NIH Animal Care and User Committee (ACUC), with approval ID ASP-LMVR5.

Note that full information on the approval of the study protocol must also be provided in the manuscript.

Plants

Seed stocks	N/A
Novel plant genotypes	N/A
Authentication	N/A
